# Broadband mode conversion via gradient index metamaterials

**DOI:** 10.1038/srep24529

**Published:** 2016-04-21

**Authors:** HaiXiao Wang, YaDong Xu, Patrice Genevet, Jian-Hua Jiang, HuanYang Chen

**Affiliations:** 1 College of Physics, Optoelectronics and Energy & Collaborative Innovation Center of Suzhou Nano Science and Technology, Soochow University, No. 1 Shizi Street, Suzhou 215006, China; 2 Centre de Recherche sur l’Hétéro-Epitaxie et ses Applications, CNRS, Rue Bernard Gregory, Sophia-Antipolis, 06560 Valbonne, France; 3 Key Lab of Advanced Optical Manufacturing Technologies of Jiangsu Province & Key Lab of Modern Optical Technologies of Education Ministry of China, Soochow University, Suzhou 215006, China

## Abstract

We propose a design for broadband waveguide mode conversion based on gradient index metamaterials (GIMs). Numerical simulations demonstrate that the zeroth order of transverse magnetic mode or the first order of transverse electric mode (TM_0_/TE_1_) can be converted into the first order of transverse magnetic mode or the second order of transverse electric mode (TM_1_/TE_2_) for a broadband of frequencies. As an application, an asymmetric propagation is achieved by integrating zero index metamaterials inside the GIM waveguide.

Along with the rapid development of photonic integrated circuits, the mode control techniques including mode filtering, mode separation, and mode conversion become crucial for designs of integrated optical systems. Among these techniques, mode conversion plays a more fundamental role and thus received a considerable research interest^1^,^2^,^3^,^4^,^5^,^6^,^7^,^8^,^9^. There have been several methods to achieve mode conversion. Usually, an excellent mode converter is characterized by large bandwidth, low loss and extinction ratio. For example, it has been suggested that an ultra-compact interferometer formed by nano-waveguides can function as mode converter by introducing an optical phase difference^3^. Such an ultracompact structure features low loss, but with polarization-dependence and narrow bandwidth as its shortage. In ref. 9, a similar geometry has been proposed for broadband mode conversion based on interference effect of light propagating through two differential dielectric waveguides. In both systems, the key is to recombine light after introducing a phase difference between two different channels. However, beam splitters at the beginning and at the end of such devices generally induce significant backscattering that reduces the efficiency of mode conversion. Suppressing the backscattering is therefore essential to improve the performance of interferential mode converters.

Nanostructured optical interfaces or metasurfaces have recently opened new avenues for manipulating light properties at interfaces^10^,^11^. In particular, metasurfaces based on gradient index metamaterials (GIMs) have been proposed to completely convert propagating waves into surface-like waves^12^. Waveguides with symmetric GIMs can convert the propagating mode (PM) gradually into surface-like mode (SM) with negligible scattering, achieving asymmetric propagation and waveguide cloaking for a broadband of frequencies independent of the polarization of the incident wave^13^,^14^. It is therefore desirable to utilize GIMs to design a waveguide structure for mode conversion with suppressed backscattering and high mode conversion efficiency. In this work, we will introduce asymmetric GIMs into the waveguide, and theoretically demonstrate that mode conversion between two lowest waveguide modes can be achieved for a broad bandwidth of frequencies and independent of incident polarizations.

## Results and Discussion

**Figure 1:**
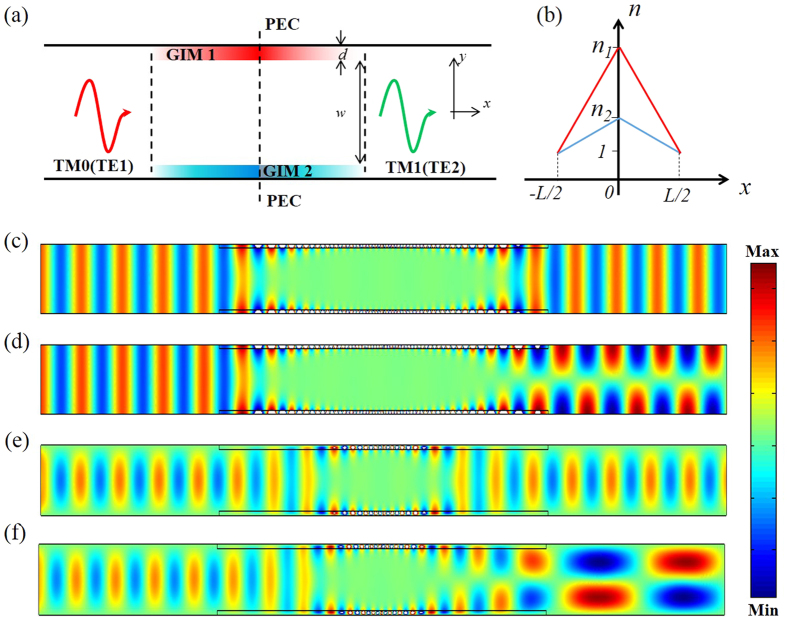
(**a**) The schematic plot of the waveguide mode converter. (**b**)The gradient index profiles of the two GIM slabs (all are larger than unity), where n_1_ is the maximum refractive index of the upper GIM slab, while n_2_ is the maximum refractive index of the lower GIM slab. (**c,d**) The simulated magnetic field patterns for TM_0_ mode incident from left to right at 9.5 GHz with (**c**) g_i_ = 1 (i = 1, 2) and (**d**) g_i_ (i = 1, 2) equal to 1.015 and 1, respectively. The output mode in (**d**) is converted into a TM_1_ mode. (**e,f**) The simulated electric field patterns for TE_1_ mode incident from left to right at 12 GHz with (**e**) g_i_ = 1 (i = 1, 2) and (**f**) g_i_ (i = 1, 2) equal to 1.018 and 1, respectively. The output mode in (**f**) is converted into a TE_2_ mode.

The schematic diagram of the waveguide with asymmetric GIMs is shown in Fig. 1a. It is a one dimensional parallel-plate waveguide with two GIMs attached to its outer perfect electric conductor (PEC) walls. The index profiles of the two GIMs along *x* direction are different from each other and can be described as


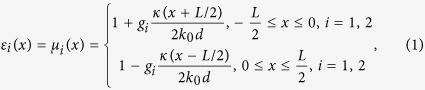


where κ = 0.2 *k*_*0*_ (*k*_*0*_ is the wave vector in free space) is a predesigned momentum parameter, *d* = 1.5 mm is the thickness of the GIMs along *y* direction. The length of the waveguide is *L* = 240 mm, *w* = 22.5 mm is the distance between the two parallel GIMs. Furthermore, *g*_*1*_ and *g*_*2*_ are referred as the gradient factors of the upper and lower GIMs, respectively. We will show that the mode conversion effect can be tuned by adjusting the values of *g*_*1*_ and *g*_*2*_ as well as the working frequencies. The difference between *g*_*1*_ and *g*_*2*_ is crucial for mode conversion. A classical geometric optics interpretation will be introduced later. The mode conversion effect is actually due to the difference of the accumulated phases during the light propagation along the upper and lower GIMs. To depict the differences between the two GIMs, their index profiles are plotted in Fig. 1b (all are larger than unity). As long as the refractive index does not vary too quickly, the waveguide structure induces negligible backscattering. For this reason we set both gradient factors around unity. In view of this configuration, the index profiles of GIMs in ref. 14 is a special case with *g*_*1*_ = *g*_*2*_ = 1.

To visualize the mode conversion effect, we perform numerical simulations by using the COMSOL Multiphysics. Both TM and TE polarizations are investigated. For TM polarization, the TM_0_ mode with a frequency of 9.5 GHz is incident from the left port. When both upper and lower GIMs have the same gradient factor (*g*_*1*_ = *g*_*2*_, i.e. they are symmetric, which has been used for waveguide cloaking in ref. 14), the output waveguide mode keeps the same as the input waveguide mode (TM_0_), as shown in Fig. 1c. However, when the gradient factor of the upper GIM *g*_*1*_ is slightly modified and the other gradient factor *g*_*2*_is kept unchanged, mode conversion from TM_0_ to TM_1_ can be achieved. Figure 1d shows that, when *g*_*1*_ and *g*_*2*_ are set as 1.015 and 1, respectively, a nearly complete waveguide mode conversion is realized at a frequency of 9.5 GHz: the output mode turns into TM_1_ mode completely. For the case of TE polarization, similar results can be found. For example, in Fig. 1e, a TE_1_ mode with a frequency of 12 GHz is incident from left, when the upper GIM slab and lower GIM slab have the same gradient factor, the same TE_1_ mode will come out from the output port. However, when the gradient factor of the upper GIM slab *g*_*1*_ is tuned to 1.018 while the gradient factor of the lower GIM slab *g*_*2*_ remains to be unity, the output mode will be converted into a TE_2_ mode. It should be noted that due to the low gradient factors, there is little backscattering for the waveguide, unless certain Fano resonances of higher modes are excited^15^, which we will discuss in the Supplementary Information 1. These results clearly demonstrate that our device operates as a mode converter for both TM and TE polarizations. Note that we are using a perfect model with the same permittivity and permeability profiles, one can also get similar performance by replacing them with normal dielectric profiles (see Supplementary Information 2 and Supplementary Fig. 4).

In the frequency region considered in this work, only two lowest modes are supported in the output port of the waveguide. Therefore the output electromagnetic wave generally consists of two parts. For TM polarization, these are the magnetic fields H_0_ and H_1_ for the zeroth order and the first order of eigenmodes, respectively. That is,


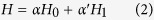


For TE polarization, they are the electric fields E_1_ and E_2_ for the first order and the second order of eigenmodes, i.e.,


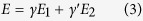


The coefficients *α, α’, γ*, and *γ’* describe the amplitudes of these eigenmodes at the output port. Thus |*α|*^2^ and *|γ|*^2^ refer to the fraction of output TM_0_ or TE_1_ mode, which is closely related to the conversion efficiency. Generally, the conversion efficiency is defined as *η = T·f*, where *T* is the transmission of both modes, and *f* is the fraction of TM_1_ or TE_2_ mode, i.e., *f* = *|α’|*^2^/(*|α|*^2^ + *|α’|*^2^) or *f* = *|γ’|*^2^/(*|γ|*^2^ + *|γ’|*^2^).Thanks to the small gradient factors, the PM can be gradually transferred into SM with suppressed backscattering, indicating that the transmission in our waveguide system is mostly close to unity.

**Figure 2:**
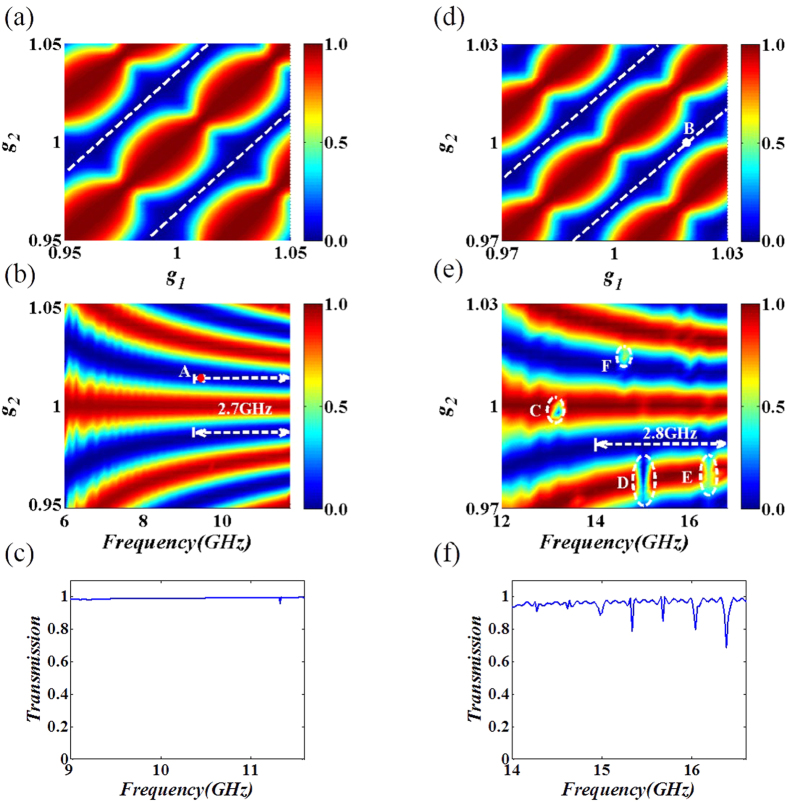
The characteristics of mode converter with different variables. (**a–c**) For TM polarization, (**a**) the changing tendency of |α|2 with different gradient factors of GIM slabs for a fixed working frequency at 6 GHz. (**b**) The changing tendency of |α|2 with different working frequencies as well as the gradient factor of the lower GIM slab. The gradient factor of the upper GIM g_1_ is fixed at 1. Point A in (**b**) corresponds to the case mentioned in Fig. 1d. Two white dash lines in (**b**) refer to the broadband mode converting regions with the fluctuation limited to 5%, where g_2_ are set as 1.015 and 0.985, respectively. The bandwidth is about 2.7 GHz. (**c**) Transmission of the case with g_1_ = 1.015, g_2_ = 1. (**d–f**) For TE polarization, (**d**) the changing tendency of |γ|^2^ with different gradient factors of GIM slabs for a fixed working frequency at 12 GHz. Point B in (**d**) corresponds to the case mentioned in Fig. 1f. (**e**) The changing tendency of |γ|^2^ with different working frequencies as well as the gradient factor of the lower GIM slab. The gradient factor of the upper GIM g_1_ in both cases is fixed at 1. One white dash line in (**e**) shows the broadband mode converting functionality for a broad bandwidth of about 2.8 GHz. Four special areas denoted by dash white circles (C,D,E and F) in (**e**) indicated the transmission dips caused by Fano resonances. (**f**) Transmission of the case with g1 = 1, g_2_ = 0.99.

In the following, we show how to continuously tune output mode as function of the frequency by adjusting both gradient factors *g*_*1*_ and *g*_*2*_. In order to explore the parameter space through which mode conversion efficiently happens, the values of |*α|*^2^ and *|γ|*^2^ are numerically calculated for different variables, such as the gradient factors of GIMs and the working frequencies. Firstly, let us explore the case of TM polarization. Figure 2a shows the varying fraction of the output TM_0_ mode along with the varying gradient factors of the two GIM slabs, where the working frequency is fixed at 6 GHz. It is consistent with intuition that either changing *g*_*1*_ or *g*_*2*_ have the same effects, as clearly shown in Fig. 2a. The diagram is symmetric along the diagonal line. Two dash lines in the blue regions refer to the contour line of *|α|*2 equal to zero, i.e., there is no TM_0_ mode in the output port (all of them have been converted to TM_1_ mode). Moreover, we observed that the mode converter works over a broad range of frequencies. Figure 2b shows the relationship between the fraction of the output TM_0_ mode and the gradient factor of the lower GIM slab (*g*_*2*_) as well as the working frequencies by fixing *g*_*1*_ = 1. The diagram appears to be periodic along with the varying gradient factor of the lower GIM slab. Point A refers to the case mentioned in Fig. 1d, which indicated that the output mode has been converted into TM_1_ mode completely. As the frequency increases, the fraction of the output TM_0_ mode tends to change more slowly. In particular, when *g*_*2*_ is fixed at 1.015 or 0.985, and the working frequencies range from 9 GHz to 11.7 GHz, the fraction of output mode *|α|*2 tend to be a nearly-constant and close to zero, as revealed by the two white dash lines in Fig. 2b. Hence, the working frequencies have a 26% bandwidth (2.7 GHz). For details, we plot the transmission curve for *g*_*1*_ = 1.015, and *g*_*2*_ = 1 in Fig. 2c for a finer frequency resolution, where we find that indeed high transmission happens for a broad band of frequencies. One may extend the converting frequency to a higher value, however, it should be noted that higher order modes will be excited for both ports, which will influence the conversion efficiency and more physical scenarios should be taken into consideration.

As for TE polarization, similar effects are observed, but compared with TM_0_ mode, TE_1_ mode has higher working frequencies in the same waveguide structure. Therefore, we limit the gradient factors range from 0.97 to 1.03 and keep the working frequency range from 12 GHz to 16.8 GHz, ensuring that the output port can only support TE_2_ mode but no higher order modes. Figure 2d presents the relationships between the fraction of output TE_1_ mode and varying gradient factors of the two GIM slabs, which also appears to be periodic and symmetric. Point B refers to the case mentioned in Fig. 1f, which indicated that the output mode has been converted into TE_2_ mode completely. In order to explore whether such a mode converter works for a broadband of frequencies, we plot the fraction of output TE_1_ mode with varying working frequencies and the gradient factor of the lower GIM slab (*g*_*2*_) by fixing g_1_ = 1, as shown in Fig. 2e. For *g*_*2*_ = 0.99, we find that the fraction of output mode |*γ|*^2^ tends to be a nearly-constant and close to zero, as revealed by the white dash line. The working frequencies now range from 14 GHz to 16.8 GHz, showing an 18% bandwidth (2.8 GHz). We then plot the transmission from 14 GHz to 16.8 GHz for the case of *g*_*1*_ = 1 and *g*_*2*_ = 0.99 in Fig. 2f for a finer frequency resolution. Except for several weak dips, the transmission is high for a broadband of frequencies. Even for the frequencies of dips, the transmission is above 0.7; therefore the effect will not be compromised too much.

There are several special areas denoted by white dash circles in the diagram, which shows that there are some strong transmission dips. Such dips come from Fano resonances, which we will discuss in details in the Supplementary Information 1 (see Supplementary Figs 1–3). In spite of these special areas, broadband mode converter via GIMs without polarization limitation is still clearly verified.

**Figure 3:**
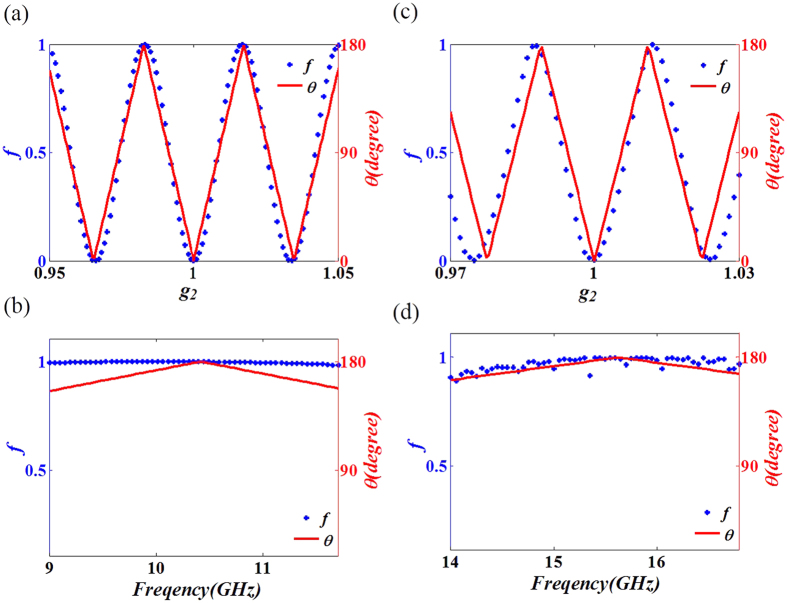
The illustration of accumulated phase difference and the fraction of output mode with varying gradient factors and working frequencies. (**a,b**) For TM polarization, red solid line refers to the accumulated phase difference between two GIM slabs, blue star data points refer to the fraction of TM_1_ mode, (**a**) with g_1_ and the working frequency fixed at 1 and 6 GHz respectively, (**b**) with g_1_ and g_2_ fixed at 1.015 and 1, i.e., the set-up of broadband converter for TM polarization. (**c,d**) For TE polarization, red solid lines refer to the accumulated phase difference between two GIM slabs, blue star data points refer to the fraction of TE_2_ mode, with g_1_ and working frequency fixed at 1 and 12 GHz, (**d**) with g_1_ and g_2_ fixed at 1 and 0.99, i.e., the set-up of broadband converter for TE polarization. Both (**a,c**) and (**b,d**) demonstrate that mode conversion is essentially controlled by the accumulated phase difference between the two asymmetric GIM slabs.

Now, let’s examine the mechanism behind the proposed mode converter. As mentioned in ref. 13 and ref. 14, the GIM slabs can convert a PM to a SM with a nearly 100% efficiency, causing little scattering. This can be achieved because the band branch of TM_0_/TE_1_ mode goes below the light line as the refractive indexes of dielectrics of GIM slabs increase (see Fig. 2e,f of ref. 13). Here for the mode converter, similar physics happens. The only difference is that owing to the asymmetric gradient factors, there is a phase difference of surface modes in the upper and lower GIM slabs. For simplicity, the wave vector of SM (*β*) at a specific position of each GIM slab is approximately equal to the wave vector in a bulk media with a dielectric constant equal to that of each GIM slab at the position, which can be viewed as *β = *2*πf·n(x)/c*. The changing of refractive index *n* gives rise to different wave vectors and consequently, different accumulated phases during light propagation in the upper and lower GIM slabs respectively. The accumulated phase difference between these two GIM slabs can be defined as 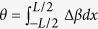. If *θ* equal to 2*nπ (n* = 0, 1, 2…), there is no phase accumulations between upper and lower GIM slabs, i.e. the output mode keep the same as the input mode (TM_0_ or TE_1_ mode). While if *θ* equal to (2*n + 1)π* (n = 0, 1, 2…), the optical field through the upper GIM has opposite sign relative to the optical field through the lower GIM. At this condition the output mode turn to the higher mode (TM_1_ or TE_2_ mode). Figure 3 represents the fraction of output mode (TM_1_ and TE_2_) and the phase difference *θ* as a function of *g*_*2*_ with a fixed *g*_*1*_ and fixed working frequency, or as a function of working frequency with *g*_*1*_ and *g*_*2*_.

In order to show the periodically changing fraction of output mode and the phase difference in the same figure, we limit the phase difference from 0 to 180 degree. Note that both TE and TM mode conversion can be explained with the same physical mechanism, we discuss TM polarization only and list the results of TE polarization without detailed explanations. As show in Fig. 3a, when there is no accumulated phase difference (i.e. *θ* equal to 0 degree), the fraction of the output TM_1_ mode keeps its minimum value thus no mode conversion occurs, while if the accumulated phase difference reach to its peak (i.e. *θ* equal to odd integer multiply of *π*), the fraction of output TM_1_ mode increased to unity, meaning that mode conversion is maximized. It should be emphasized that this explanation is applicable to the case that both gradient factors are around unity. Otherwise, the explanation may not function very well, as also revealed in Fig. 3a. When *g*_*2*_ equals to 0.95 or 1.05, the result is not so exact as that when *g*_*2*_ equals to unity. Regardless of it, both two parameters agree with each other very well with the varying gradient factor. It verifies our explanation that the changing of output mode is stemmed from the asymmetric GIM slabs, which can approximately explained by the accumulated phase difference between these two GIM slabs. The same phenomenon is found for TE polarization (see in Fig. 3c). We then fixed *g*_*1*_and *g*_*2*_, and plot the accumulated phase difference and the fraction of the output mode at different working frequencies in Fig. 3b (TM) and 3d (TE), for the broadband mode converter mentioned in Fig. 2c,f. We find that the fraction of the output mode coincides with the accumulated phase difference very well for a broadband of frequencies for both polarizations. This also explain why the mode converter can work in a broadband of frequencies. As both gradient factors are very close to unity, the accumulated phase differences will not deviate too much from *π* for the working frequencies. However, we should emphasize that the accumulated phase difference method is only approximately correct. When the distance between the two GIMs becomes smaller, the coupling effect will be stronger and the mode conversion will be compromised.

**Figure 4:**
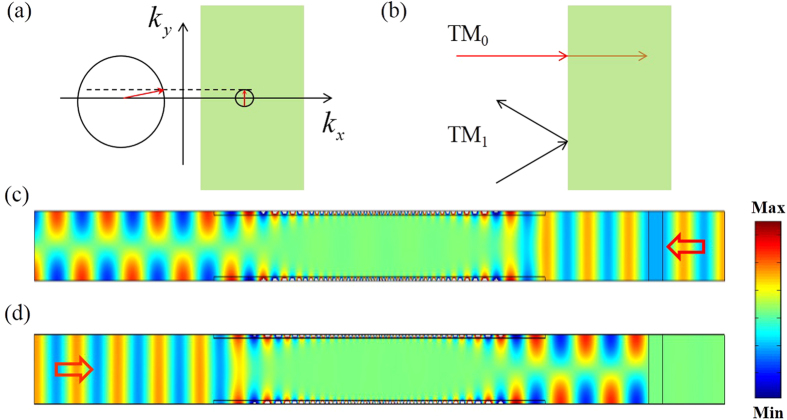
Asymmetric propagation based on waveguide mode conversion coupled with ZIMs. (**a,b**) The schematic diagram of reflection and refraction at the interface of air and ZIMs (indicated by green areas). (**c,d**) The simulated magnetic field patterns for TM_0_ wave in the waveguide structure with ZIMs, (**c**) refers to the case where the wave is incident from right to left, while (**d**) refers to that from left to right. Other parameters are set as follows: g_1_ = 1.015, g_2_ = 1, and f = 9 GHz.

As an application, we combine the properties of our mode converter with those of a slab of zero index metamaterials (ZIMs) to achieve asymmetric propagation in a waveguide. ZIMs have many interesting properties. For example, they can be used to enhance the directive emission for an embedded source^16^, or to squeeze electromagnetic waves in a narrow waveguide with ZIMs^17^,^18^. In addition, total transmission and total reflection can be achieved by introducing defects in ZIMs^19^,^20^,^21^. Figure 4a,b schematically show the transverse momentum conservation for reflection and transmission at the interface air/ZIMs. The bigger circle represents the isofrequency curve in air while the smaller one denotes that in ZIMs. The red arrow in the bigger circle is at the critical incident angle, which is nearly horizontal. For the incident angle larger than the critical angle, the ray cannot transmit into ZIMs and has a total reflection at the interface. To be more precise, when the TM_0_ mode is incident on a ZIM surface, it will propagate through ZIMs as it was a transparent media with a zero reflection coefficient. While TM_1_ mode are totally reflected, as shown in Fig. 4b. Therefore, putting a ZIM block into the right port of our waveguide structure together with the asymmetric GIM slabs, we achieve asymmetric propagation for TM polarization. Simulations are performed to demonstrate the asymmetric propagation, the related parameters are set as follow: *g*_*1*_ = 1, *g*_*2*_ = 1.015, and working frequency is 9 GHz. As shown in Fig. 4c, when the TM_0_ mode with a frequency of 9 GHz is incident from the right port, it will first pass through the ZIM and then continue to propagate in the GIM waveguide, leaving the left port as a TM_1_ mode. However, when the TM_0_ is incident from the left port, it will first be converted into a TM_1_ mode after passing through the GIM part. Then it experiences total reflection after reaching the ZIM part, demonstrating asymmetric propagation as shown in Fig. 4d.

## Conclusions

In this paper, we have proposed a broadband mode converter which consists of a waveguide with two slabs of gradient index metamaterials coated on its sides. We have studied numerically and analytically the efficiency of mode conversion from TM_0_/TE_1_ into TM_1_/TE_2_ as function of metamaterial gradient factors and working frequencies. In particular, we have shown that our device can maintain relatively high mode conversion over a broad range of frequencies due to the slowly varying refractive index profiles of gradient index metamaterials. We have explained, using simple arguments, that the accumulated phase difference caused by light propagation in the asymmetric GIMs controls waveguide mode conversion. In addition, we have proposed an interesting application of asymmetric TM propagation by introducing zero index metamaterials at one port of the GIM waveguide.

## Methods

The field patterns in Fig. 1(c–f) and Fig. 4(c,d) and the transmissions in Fig. 2(c,e), as well as the calculated fraction of TE_1_ and TM_0_ in Fig. 3(a–d) were obtained by using the finite element solver COMSOL Multiphysics. Ports were set as boundary conditions of both sides of waveguides to obtain the transmission.

## Additional Information

**How to cite this article**: Wang, H.X. *et al*. Broadband mode conversion via gradient index metamaterials. *Sci. Rep*. **6**, 24529; doi: 10.1038/srep24529 (2016).

## Supplementary Material

Supplementary Information
