# Supplementary material for: Broadband mode conversion via gradient index metamaterials
Source: Sci Rep. 2016 Apr 21;6:24529. doi: 10.1038/srep24529 (PMC4838908; doi:10.1038/srep24529)
Supplement: Supplementary Information [file srep24529-s1.doc]

**Supplementary Information for:**

**Broadband mode conversion via gradient index metamaterials**

**HaiXiao Wang^1,+^, YaDong Xu^1,+ , *^, Patrice Genevet ^2, *^ , Jian-Hua Jiang ^1, *^, HuanYang Chen ^1, 3, *^**

*^1^ College of Physics, Optoelectronics and Energy & Collaborative Innovation Center of Suzhou Nano Science and Technology, Soochow University, No.1 Shizi Street, Suzhou 215006, China*

*^2^ Centre de Recherche sur l’Hétéro-Epitaxie et ses Applications, CNRS, Rue Bernard Gregory, Sophia-Antipolis, 06560 Valbonne, France.*

*^3^ Key Lab of Advanced Optical Manufacturing Technologies of Jiangsu Province & Key Lab of Modern Optical Technologies of Education Ministry of China, Soochow University, Suzhou 215006, China*

^*^ ydxu@suda.edu.cn; patrice.genevet@crhea.cnrs.fr; joejhjiang@sina.com; chy@suda.edu.cn

^+^These authors contributed equally to this work

**1. Special cases caused by Fano resonances**

For some cases of TE polarization, the system exhibits strong Fano resonances. For example, for "C", "D", "E" and "F" in Fig. 2e, the transmission reaches to values near zero. We plot the transmission for the case of "C", "D", "E", and "F" in Supplementary Fig. 1(a-d), respectively. We find that such cases show clear asymmetric resonant line shapes, i.e. they come from Fano resonances.


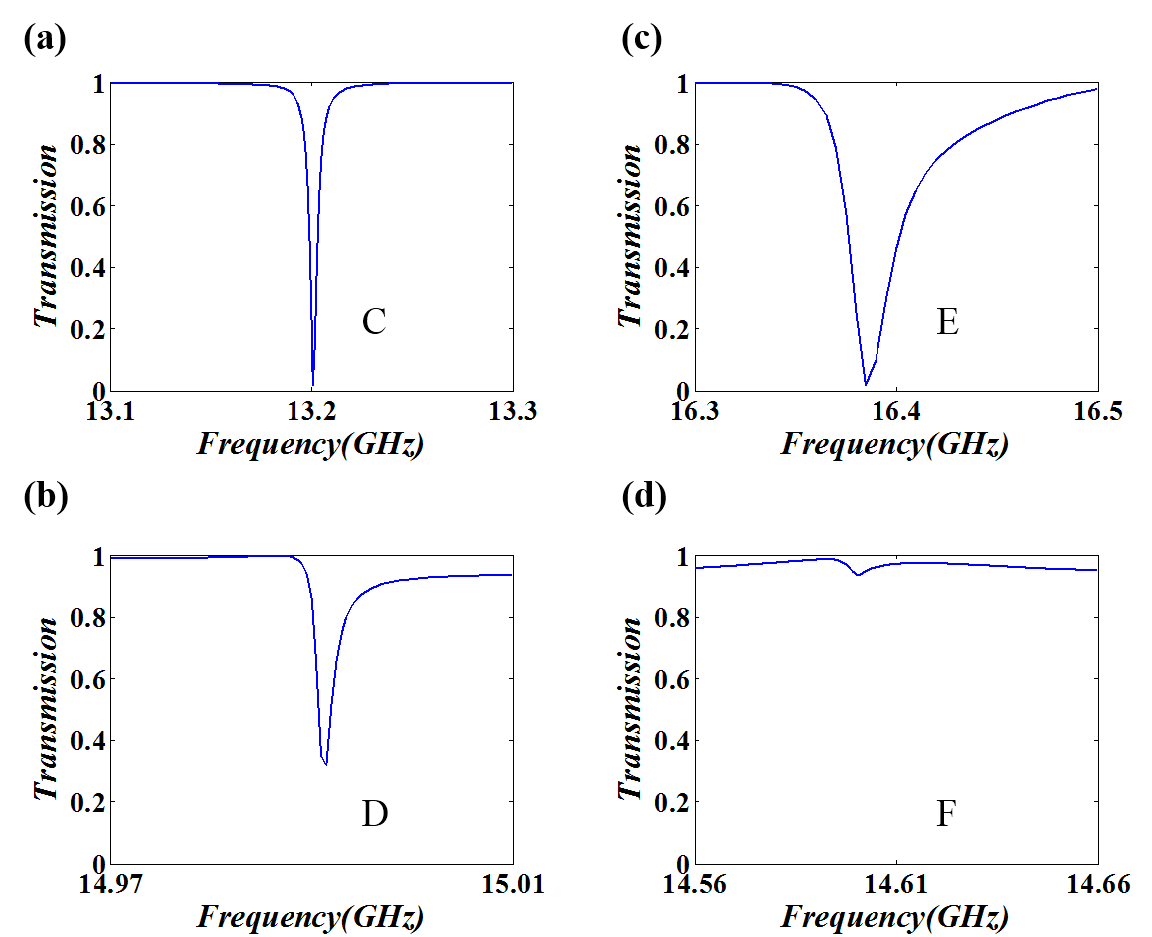


***Supplementary Figure 1****. Transmission of the waveguide with different gradient factors for TE polarization, (a) for the case of "C" in Fig. 2e, (b) for the case of "D" in Fig. 2e, (c) for the case of "E" in Fig. 2e, (d) for the case of "F" in Fig. 2e.*

Let us first examine the field patterns at the above transmission dips in Supplementary Fig. 2, where we find that all of these field patterns share the same characteristic: higher order modes are excited at the middle part of the waveguide and large amount of energy is confined in the area of waveguide with GIM slabs.


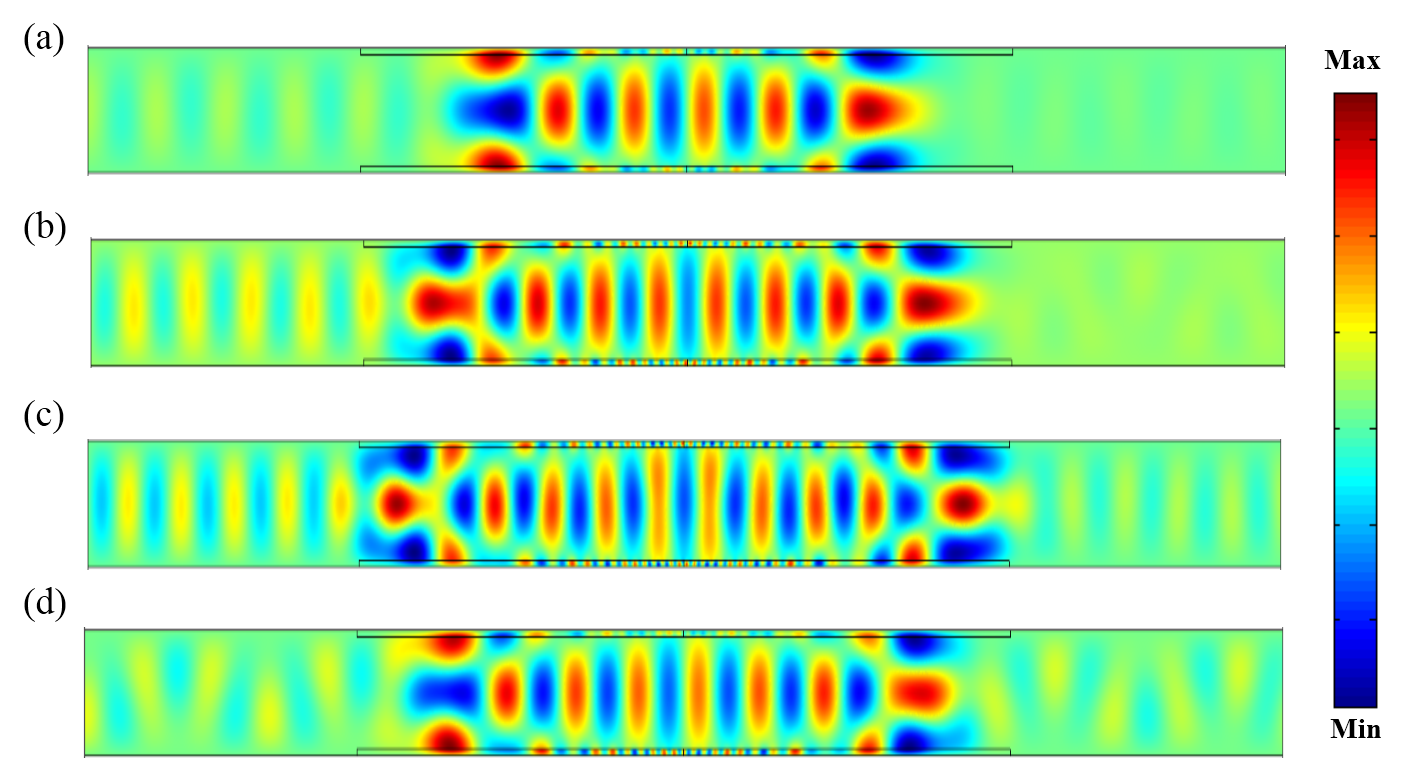


***Supplementary Figure 2****. Electric field patterns of different cases, (a) refer to case "C" at the resonant frequency of 13.2GHz, (b) refer to case "D" at resonant frequency of 15GHz, (c) refer to case "E" at resonant frequency of 16.4GHz, (d) refer to case "F" at resonant frequency of 14.6GHz.*

To explore further, we plot the dispersions of case "C" mentioned above in Supplementary Fig. 3 (with symmetric GIMs) to explain the underlying physics.


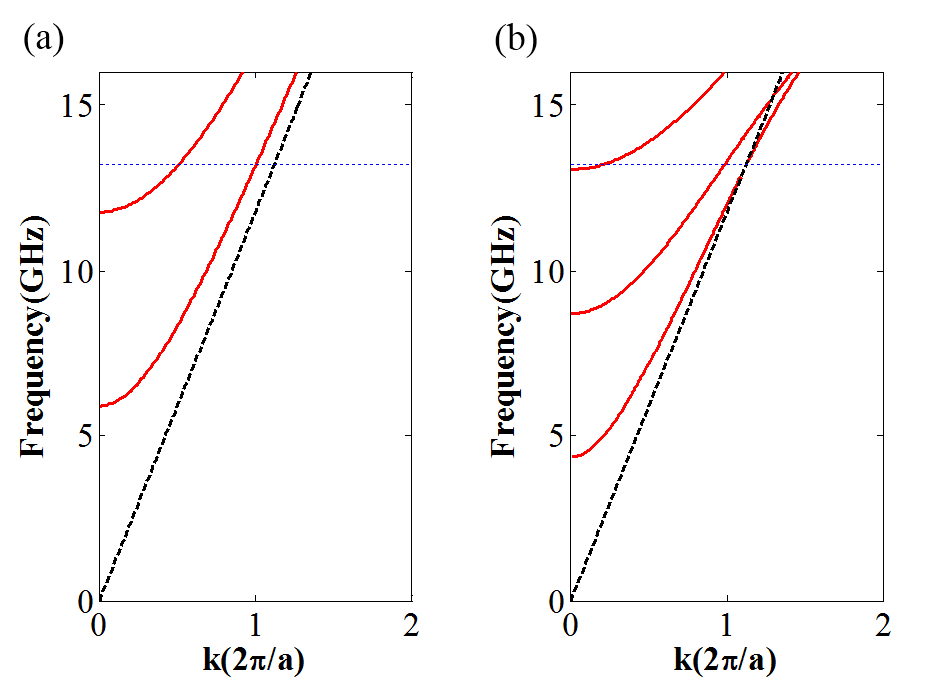


***Supplementary Figure 3*** *The dispersion relations for TE mode with the GIMs replaced by different dielectrics. (a) The dispersion relation for TE mode by setting the refractive index of the dielectrics as 1, i.e. an empty waveguide. (b) The dispersion relation for TE mode by setting the refractive index of the dielectrics as 4. Both red solid curves refer to different modes supported in the waveguide, black dash lines are the light lines, and dash blue lines refer to the working frequencies of 13.2GHz.*

As shown in Supplementary Fig. 3, when the TE wave with a frequency of 13.2GHz incident into the waveguide, both TE_1_ and TE_2_ modes can be supported. However, due to the symmetry of the system, the TE_2_ mode (asymmetric mode) could not be excited and there is only TE_1_ mode in the waveguide. When this TE_1_ mode continues to propagate through the area of the waveguide with higher dielectrics, the dispersion is totally different, as shown in Supplementary Fig. 3(b). The waveguide can now support higher symmetric mode (TE_3_ mode), which has already been found in the field patterns in Supplementary Fig. 2. Thus, two symmetric modes (TE_1_ and TE_3_ modes) coexist in the area of the waveguide with dielectrics. However, we should keep in mind that TE_3_ does not always exist in the whole waveguide structure. As the refractive index of the dielectrics turns to be lower, TE_3_ mode cannot transmit through the output ports of waveguide. In fact, TE_3_ mode will experience an open cavity and emit energy in forms of TE_1_ mode at resonances. This part of energy will interfere with original TE_1_ mode that already propagate in the waveguide system, forming Fano resonances, see more details in Ref. 15.

Hence the above sharp dips for the special areas denoted by white dash circles in Fig. 2e come from Fano resonances. In fact, the weak dips in Fig. 2f also share the same physics, and the resonance becomes stronger and stronger, as the frequency increases.

**2 Mode converter with normal dielectric profiles**

By keeping the refractive indexes unchanged and letting *μ_i_*=1(*i*=1, 2), we can obtain normal dielectric profiles with

$\sqrt{\varepsilon_{i}(x)}=\left\{\begin{matrix} 1+g_{i}\frac{\kappa(x+L/2)}{2k_{0}d},-\frac{L}{2}\leq x\leq0,i=1,2 \\ 1-g_{i}\frac{\kappa(x-L/2)}{2k_{0}d},0\leq x\leq\frac{L}{2},i=1,2 \end{matrix}\right.,$ (S1)

which can be utilized to realize a mode converter. For incidence, we use dielectric profiles with the same sizes of Fig. 1d, to convert a TM_0_ mode to a TM_1_ mode, as plotted in Supplementary Fig. 4(a). We can also use dielectric profiles with the same sizes of Fig. 1f, to convert a TE_1_ mode to a TE_2_ mode, as plotted in Supplementary Fig. 4(b). It should be noted that both cases are not optimization designs, thus inducing some backscattering. Nevertheless, such devices can be easily realized in microwave frequencies following the method in Refs.13-15.


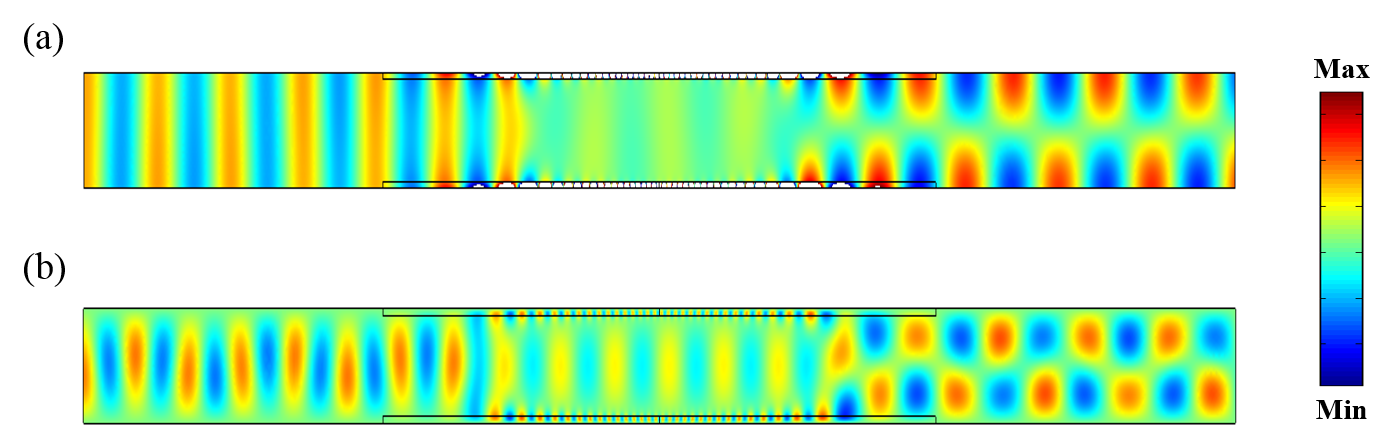


***Supplementary Figure*** ***4*** *The mode converter with normal dielectric profiles. (a) The simulated field patterns for TM_0_ mode incident from left to right at 9.5GHz with g_i_=1(i=1, 2) equal to 1.015 and 1, respectively. The output mode converted into TM_1_ mode. (b) The simulated field patterns for TE_1_ mode incident from left to right at 14.3GHz with g_i_=1(i=1, 2) equal to 1.01 and 1, respectively. The output mode converted into TE_2_ mode.*
